# The Neuroprotective Effect of L-Carnitine against Glyceraldehyde-Induced Metabolic Impairment: Possible Implications in Alzheimer’s Disease

**DOI:** 10.3390/cells10082109

**Published:** 2021-08-17

**Authors:** Simona Magi, Alessandra Preziuso, Silvia Piccirillo, Francesca Giampieri, Danila Cianciosi, Monia Orciani, Salvatore Amoroso

**Affiliations:** 1 Department of Biomedical Sciences and Public Health, School of Medicine, University “Politecnica delle Marche”, Via Tronto 10/A, 60126 Ancona, Italy; a.preziuso@pm.univpm.it (A.P.); s.piccirillo@staff.univpm.it (S.P.); s.amoroso@staff.univpm.it (S.A.); 2 Department of Clinical Sciences, School of Medicine, University “Politecnica delle Marche”, Via Tronto 10/A, 60126 Ancona, Italy; f.giampieri@staff.univpm.it (F.G.); danila.cianciosi@gmail.com (D.C.); 3 Department of Biochemistry, Faculty of Sciences, King Abdulaziz University, Jeddah 21577, Saudi Arabia; 4 Department of Clinical and Molecular Sciences-Histology, School of Medicine, University “Politecnica delle Marche”, Via Tronto 10/A, 60126 Ancona, Italy; m.orciani@staff.univpm.it

**Keywords:** Alzheimer’s disease, L-carnitine, metabolic dysfunctions, mitochondrial membrane potential, neuronal survival, oxygen consumption rate, oxidative stress, Tau hyperphosphorylation

## Abstract

Alzheimer’s disease (AD) is a neurodegenerative disorder characterized by progressive cognitive regression and memory loss. Dysfunctions of both glucose metabolism and mitochondrial dynamics have been recognized as the main upstream events of the degenerative processes leading to AD. It has been recently found that correcting cell metabolism by providing alternative substrates can prevent neuronal injury by retaining mitochondrial function and reducing AD marker levels. Here, we induced an AD-like phenotype by using the glycolysis inhibitor glyceraldehyde (GA) and explored whether L-carnitine (4-N-trimethylamino-3-hydroxybutyric acid, LC) could mitigate neuronal damage, both in SH-SY5Y neuroblastoma cells and in rat primary cortical neurons. We have already reported that GA significantly modified AD marker levels; here we demonstrated that GA dramatically compromised cellular bioenergetic status, as revealed by glycolysis and oxygen consumption rate (OCR) evaluation. We found that LC ameliorated cell survival, improved OCR and ATP synthesis, prevented the loss of the mitochondrial membrane potential (Δψ_m_) and reduced the formation of reactive oxygen species (ROS). Of note, the beneficial effect of LC did not rely on the glycolytic pathway rescue. Finally, we noticed that LC significantly reduced the increase in pTau levels induced by GA. Overall, these findings suggest that the use of LC can promote cell survival in the setting of the metabolic impairments commonly observed in AD. Our data suggest that LC may act by maintaining mitochondrial function and by reducing the pTau level.

## 1. Introduction

Alzheimer’s disease (AD) is a neurodegenerative disorder characterized by progressive cognitive regression and memory loss. AD is defined by the presence of specific markers in the cerebral cortex, namely, neuritic plaques, which are mostly composed of fibrillary amyloid-β (Aβ), and abundant neurofibrillary tangles, which are composed of hyperphosphorylated Tau protein (pTau) [1,2]. The complexity of the pathogenic framework that characterizes AD has limited the development of effective mechanism-based therapies. Different cofactors may converge on AD pathogenesis and shape its course [1,3]. In particular, the dysfunction of glucose metabolism, and the alteration of mitochondrial dynamics and oxidative stress have been recognized as the main upstream events of the degenerative processes leading to AD [4,5]. The disruption of glucose metabolism has been shown to affect the production and clearance of Aβ and Tau phosphorylation [6], events that may occur as a consequence of both the depletion of ATP and the production of advanced glycation end products (AGEs) [6,7,8], which in turn may favor ROS formation and worsen mitochondrial performance. In this framework, a decrease in the mitochondrial membrane potential (Δψ_m_) has also been observed in both AD animal models and in human cortical neurons ex vivo [9,10,11]. Interestingly, a link between the hyperphosphorylation of Tau, oxidative stress and mitochondrial dysfunctions has been proposed [12], as the hyperphosphorylation of Tau protein may be the earliest event occurring during abnormal Tau processing in AD and other Tau pathologies [13,14]. In this scenario, it has recently been found that correcting cell metabolism with alternative substrates, such as glutamate, has a positive impact on mitochondrial function, mainly with regard to ATP synthesis and ROS formation, leading to improved neuronal survival, which occurs in parallel with the reduction of AD markers [15].

L-carnitine (4-N-trimethylamino-3-hydroxybutyric acid, LC) is a naturally occurring compound whose primary function within the cell is to facilitate the transport of activated long-chain fatty acids into the mitochondrial matrix (the “carnitine shuttle”) before they undergo β-oxidation, resulting in ATP formation [16]. In neuronal cells, the translocation of the acetyl moiety from the mitochondria into the cytosol through the carnitine shuttle promotes the synthesis of acetylcholine and acetylcarnitine (ALC) [16,17], which, in turn, may contribute to the modulation of brain energy metabolism, synaptic morphology and synaptic transmission [16,18,19]. Interestingly, disturbances in the biosynthesis and metabolism of LC have been documented in AD patients [20,21], suggesting that the perturbed transport of fatty acids into the mitochondria for β-oxidation may contribute to the impairment of energy metabolism observed in this neuropathological setting. In this vein, early studies reported that the administration of ALC (which has greater bioavailability than LC) to AD patients [22,23] could improve cognitive functions and delay the progression of mental decline [24,25,26]. Although these findings have been corroborated by more recent studies [22,27], the underlying cellular and molecular mechanisms have not been fully elucidated. Of note, several studies reported that LC can exert neuroprotective effects in different neuropathological settings, and the mechanisms involved in these effects include either the compensation and enhancement of specific mitochondrial metabolic pathways, or the improvement of antioxidant defenses [28,29,30,31,32,33,34,35].

Considering that glucose metabolism impairment is a crucial event in the pathogenesis of AD, in the present study, we challenged neuronal cells with glyceraldehyde (GA), a glycolysis inhibitor able to cause AD-like alterations in diagnostic marker levels [15,36], and explored whether the administration of LC as an alternative substrate could sustain neuronal survival [15]. We investigated the mechanisms underlying the possible neuroprotective effect of LC, with a particular focus on the modulation of mitochondrial activities and the possible implications in AD pathology.

## 2. Materials and Methods

### 2.1. Cell Culture and Treatments

The human neuroblastoma cell line SH-SY5Y was obtained from American Type Culture Collection (CRL-2266). SH-SY5Y cells were cultured as a monolayer and grown in polystyrene dishes (100 mm diameter) in Dulbecco’s Modified Eagle’s Medium (DMEM; Corning, New York, NY, USA) supplemented with 10% fetal bovine serum (FBS), 100 U/mL penicillin, and 100 μg/mL streptomycin (Corning) and were maintained in a humidified incubator at 37 °C in a 5% CO_2_ atmosphere.

Primary rat cortical neurons were prepared from the cortex of Wistar rat pups (P2–P4) (Charles River, Lecco, Italy) as previously described [15,37]. The experimental procedures were performed in full compliance with the Committee for Animal Welfare of the University “Politecnica delle Marche” (project 40A31N.G22) and in strict accordance with the guidelines of the Italian Ministry of Health (D.L. 26/2014). All efforts were made to minimize the number of animals used as well as reducing their suffering. Briefly, the cortex was rapidly removed and placed in ice-cold PBS, and the tissue was trypsinized (0.05% trypsin/EDTA) for 15 min at 37 °C, homogenized and plated on poly-D-lysine-coated plates. Cultures were maintained at 37 °C in a humidified atmosphere of 5% CO_2_ in Neurobasal medium (Gibco-Invitrogen, Paisley, UK) supplemented with B27 (Gibco-Invitrogen) and 2 mM glutamine in the presence of penicillin/streptomycin. The medium was changed twice a week, and experiments were performed between 10 and 16 DIV (days in vitro). To estimate the protective effect of LC against GA-induced toxicity, LC (Sigma-Aldrich, St. Louis, MO, USA) was added at a final concentration of 3 mM 1 h before the addition of GA (Santa Cruz Biotechnology, Dallas, TX, USA). LC was kept in contact with the cells throughout the whole period of GA exposure. After 24 h, the cells were harvested for further analysis.

### 2.2. Cell Viability Assay

The 3-(3,4-dimethylthiazol-2-yl)-2,5-diphenyltetrazolium bromide (MTT) assay measured cell viability by assessing the ability of mitochondria to metabolize the yellow tetrazolium salt MTT to purple insoluble crystals of formazan [38,39]. Both SH-SY5Y and rat primary cortical neurons were plated on 12-well plates, and at the end of the experimental procedure, the cells were incubated with 0.5 mL of MTT solution (0.5 mg/mL in PBS) in the dark at 37 °C and a 5% CO_2_ atmosphere in a humidified incubator. After 1 h, the cells were washed with PBS, and the produced formazan crystals were dissolved in 0.5 mL of DMSO [15,40]. A decrease in mitochondrial activity resulted in a reduction in the amount of formazan produced and therefore in the absorbance value. Absorbance was read at a wavelength of 540 nm using a Victor Multilabel Counter plate reader (Perkin Elmer, Waltham, MA, USA). The results were expressed as percentages of the control value.

### 2.3. ATP Assay

The intracellular ATP content was evaluated by using a commercially available luciferase-luciferin system (ATPlite, Perkin Elmer) as previously described [15,40]. Briefly, SH-SY5Y cells were plated on a 96-well “View Plate” (Perkin Elmer), subjected to specific treatments in culture medium and then analyzed for the ATP content according to the manufacturer’s protocol. Rat cortical neurons were plated on 12-well plates, and after the specific treatments, the neurons were appropriately lysed. Thereafter, 100 μL of the lysates were transferred to a 96-well View Plate to perform the ATP assay. The ATP levels were analyzed with a luminescence Victor Multilabel Counter plate reader (Perkin Elmer), normalized to the respective protein content, and expressed as percentages of the control value.

### 2.4. Bioenergetic Analysis

Seahorse XF24 Extracellular Flux Analyzer (Seahorse Bioscience, North Billerica, MA, USA) was used to detect oxygen consumption rate (OCR) and extracellular acidification rate (ECAR), representing oxidative phosphorylation and glycolysis, respectively, as previously described [41,42,43]. Oligomycin (3 μg/mL), the uncoupler 2,4-dinitrophenol (DNP) (0.3 mM), and rotenone/antimycin A (1 μM and 2.5 μM, respectively) were sequentially introduced to measure basal respiration, maximal respiration, ATP production, spare respiratory capacity, and proton leak [42]. For ECAR, sequential injections of 1 μM rotenone, 30 mM glucose, and 100 mM 2-deoxyglucose were used to measure glycolysis, glycolytic capacity and to allow the estimation of glycolytic reserve [42].

Rat primary cortical neurons (100,000 cells/well) were seeded on poly-D-lysine pre-coated XF24 cell culture plates (Seahorse Bioscience), and subjected to the experimental protocol. Then, Neurobasal medium was replaced with 500 μL/well of XF24 running media. The plates were pre-incubated at 37 °C for 1 h in the XF Prep Station incubator (Seahorse Bioscience) in the absence of CO_2_, and then run on the XF24 analyzer to obtain OCR or ECAR.

OCR and ECAR were recorded during specified programmed time periods as the average numbers between the injections of inhibitors mentioned above. The final data calculation was performed after the readings and was normalized for total cells/well. To this purpose, cells in each well were trypsinized, collected and counted by Tali™ Image-Based Cytometer (Invitrogen, Milan, Italy). Since several factors may contribute to bioenergetics variation of primary cell cultures (e.g., cell distribution in the wells relative to where the O_2_ sensor measures, and cell survival) OCR and ECAR parameters were presented as a percentage of the respective control [41].

Glycolysis was also evaluated by using the Glycolysis Cell-Based Assay Kit (Cayman Chemical, Ann Arbor, MI, USA) [44]. The kit provided a colorimetric method to detect L-lactate, the end product of the glycolysis, produced and secreted by the cultured cells. The lactate dehydrogenase enzyme catalyzed the reaction between NAD^+^ and lactate, yielding pyruvate and NADH. The NADH directly reduced a tetrazolium salt to a colored formazan, which absorbed between 490 and 520 nm. The quantity of formazan produced is proportional to the quantity of lactate in the culture medium, thus providing an indirect measurement of glycolysis [45]. Rat primary cortical neurons were seeded on poly-D-lysine-coated 12-well plates at a density of 100,000 cells/well and subjected to the experimental protocol. The samples were analyzed according to the instructions provided by the manufacturer. Lactate concentrations were calculated according to the equation [Absorbance—(y-intercept)/Slope] × 1, then the obtained data were expressed as percentages of the control value.

### 2.5. Analysis of the Mitochondrial Inner Membrane Potential (Δψ_m_)

The Δψ_m_ was monitored by using the fluorescent probe tetramethylrhodamine ethyl ester (TMRE, Abcam, Cambridge, UK) in nonquenching mode [45,46,47]. Since dye aggregation and quenching in mitochondria did not occur, in nonquenching mode, depolarized (less negative) mitochondria had lower cationic dye concentrations and lower fluorescence and more polarized mitochondria had higher dye concentrations and fluorescence [45]. Rat primary cortical neurons were grown on poly-D-lysine-coated glass coverslips and then subjected to specific treatments. Subsequently, the neurons were loaded with 10 nM TMRE in the culture medium at 37 °C for 30 min. Then, the neurons were washed twice with PBS and transferred to a microscopy chamber in PBS in the presence of 10 nM TMRE. Confocal images were obtained using a 510 LSM microscope (Carl Zeiss, Milan, Italy) equipped with a META detection system. TMRE was excited at 543 nm, and fluorescence was measured from 580 nm to 700 nm. Images were acquired every 5 s, and the basal levels of the Δψ_m_ were monitored for approximately 300 s. Carbonyl cyanide-p-trifluoromethoxyphenylhydrazone (FCCP, 20 µM) was added at the end of each experiment as an internal control. Analysis of the fluorescence intensity was performed offline after image acquisition. The TMRE fluorescence values were reported as percentages of the control value.

### 2.6. Evaluation of Mitochondrial ROS Production

Specific evaluation of the mitochondrial ROS levels was performed by using MitoTracker CM-H2XRos (Invitrogen Life Technologies, Carlsbad, CA, USA) as previously described [15,40]. Briefly, cells were plated on coverslips (coated with poly-D-lysine for rat primary cortical neurons) and subjected to specific treatments. At the end of the experimental protocol, the cells were loaded with 300 nM dye for 30 min at 37 °C and then washed 3 times with PBS. Confocal images were obtained using a 510 LSM microscope (Carl Zeiss) equipped with a META detection system. CM-H2XRos was excited at 560 ± 10 nm, and its emission was measured at 620 ± 20 nm. Images were acquired every 5 s, and the basal levels of ROS were monitored for approximately 200 s. Analysis of fluorescence intensity was performed offline after image acquisition. The fluorescence values were reported as percentages of the control value.

### 2.7. Immunofluorescence Experiments

Rat cortical neurons were plated on poly-d-lysine-coated coverslips and subjected to specific treatments with GA and/or LC. Then, to verify the expression of the hyperphosphorylated form of Tau protein, the neurons were loaded with 300 nM MitoTracker (MitoTracker Red CMXRos M7512, Invitrogen) for 30 min at 37 °C and fixed with PBS and 3.7% formaldehyde for 30 min at RT [15]. After fixation, the cells were permeabilized with PBS and 0.1% Triton X-100 for 5 min at RT [48] and then incubated with anti-Tau AT100 (1:100; this antibody recognized phosphorylated Thr 212 and Ser 214; Thermo Fisher Scientific, Waltham, MA, USA) [49]. Immunoreactions were revealed by incubation with a conjugated secondary antibody (anti-mouse Alexa Fluor 488 dye-conjugated antibody, Thermo Fisher Scientific, dilution 1:200) for an additional 20 min. Protein fluorescence was acquired by using the LSM 510 confocal system (Carl Zeiss). Images were acquired every 5 s, and the basal fluorescence values were monitored for approximately 20 s. Analysis of fluorescence intensity was performed offline after image acquisition. The fluorescence values were reported as percentages of the control value.

### 2.8. Statistical Analysis

Data are expressed as the mean ± S.E.M. Values less than 0.05 were considered to be significant. Differences among means were assessed by one-way ANOVA followed by Dunnett’s post hoc test. Statistical comparisons were carried out by using GraphPad Prism 5 software (GraphPad Software Inc., San Diego, CA, USA).

## 3. Results

### 3.1. LC Protected SH-SY5Y Cells and Rat Primary Cortical Neurons from GA-Induced Mitochondrial Toxicity

**Figure 1:**
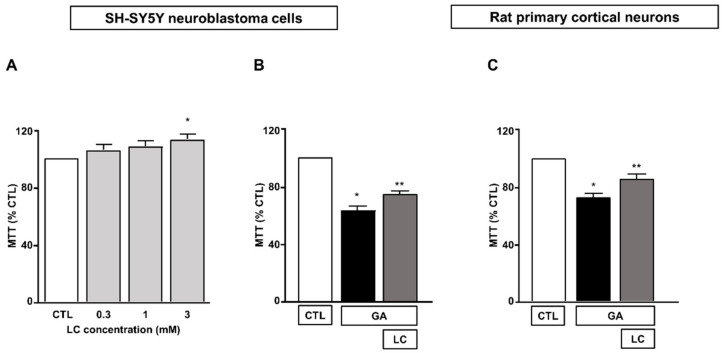
Protective effect of LC from GA-induced mitochondrial toxicity in SH-SY5Y neuroblastoma cells and in rat primary cortical neurons. (**A**) Effect of increasing concentrations (from 0.3 to 3 mM) of LC in SH-SY5Y neuroblastoma cells. Cell viability was assessed after 24 h of exposure by means of MTT assay. (**B**) Effect of LC on cell viability in SH-SY5Y neuroblastoma cells and in rat primary cortical neurons (**C**) challenged with GA. Cells were pretreated with LC (3 mM) for 1 h and then exposed to GA (1 mM) for 24 h (without removing LC). Cell viability was assessed by means of MTT assay. In each experiment, MTT reduction was expressed as a percentage of the control. Statistical differences were assessed by one-way ANOVA followed by Dunnett’s post hoc test. (**A**) F (4, 19) = 3.703. Each column represents the mean ± S.E.M. of *n* = 4–6 experiments performed in triplicate. * Significant versus CTL (*p* < 0.05). (**B**) F (2, 18) = 70.91. Each column represents the mean of *n* = 7 experiments performed in triplicate. * Significant versus all groups (*p* < 0.0001 versus CTL and *p* < 0.01 versus LC + GA); ** significant versus all groups (*p* < 0.0001 versus CTL and *p* < 0.01 versus GA). (**C**) F (2, 24) = 27.20. Each column represents the mean ± S.E.M. of *n* = 9 experiments performed in triplicate. * Significant versus all groups (*p* < 0.0001 versus CTL and *p* < 0.01 versus LC + GA); ** significant versus GA and CTL (*p* < 0.01). CTL = Control; GA = Glyceraldehyde 1 mM; LC = L-carnitine 3 mM.

We first determined the effect of 24 h of exposure to LC at concentrations ranging from 0.3 to 3 mM on SH-SY5Y neuroblastoma cells. As shown in Figure 1A, we found that under the control conditions, mitochondrial activity was significantly increased when SH-SY5Y cells were exposed to LC at a concentration of 3 mM for 24 h. Therefore, we chose to use 3 mM LC for subsequent studies. The exposure of SH-SY5Y cells to GA resulted in a significant decrease in cell viability, in line with previous findings [15,36]. A pretreatment for 1 h with 3 mM LC significantly increased the cell viability in cells challenged with GA for 24 h (without LC removal, see the “Material and Methods” for further details). This effect was observed in both SH-SY5Y cells and in rat primary cortical neurons (Figure 1B,C).

### 3.2. LC Increased the Intracellular ATP Levels and Mitochondrial Oxygen Consumption without Affecting Glycolysis in GA Challenged Cells

**Figure 2:**
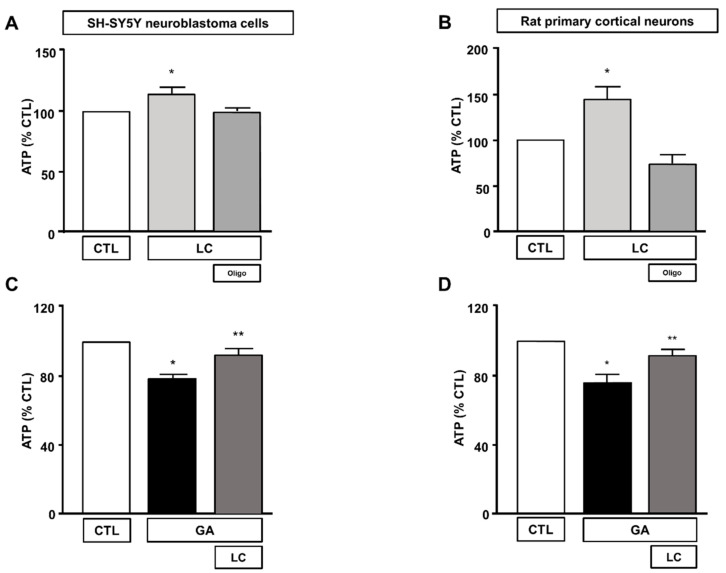
Effect of LC on intracellular ATP levels in SH-SY5Y neuroblastoma cells and in rat primary cortical neurons. Effect on intracellular ATP levels of 1 h exposure to LC under control conditions in SH-SY5Y neuroblastoma cells (**A**), and in rat primary cortical neurons (**B**) in the presence or in the absence of oligomycin (3 µg/mL). Effect of LC on intracellular ATP levels in SH-SY5Y neuroblastoma cells (**C**) and in rat primary cortical neurons (**D**) both challenged with GA. Cells were pretreated with LC (3 mM) for 1 h, and then exposed to GA (1 mM) for 24 h (without removing LC). In each experiment, ATP levels were expressed as a percentage of the control. Statistical differences were assessed by one-way ANOVA followed by Dunnett’s post hoc test. (**A**) F (3, 20) = 5.232. Each column represents the mean ± S.E.M. of *n* = 6 experiments performed in triplicate. * Significant versus all groups (*p* < 0.05). (**B**) F (3, 36) = 14.28. Each column represents the mean ± S.E.M. of *n* = 10 experiments performed in duplicate. * Significant versus all groups (*p* < 0.01 versus CTL, *p* < 0.0001 versus LC+oligo). (**C**) F (2, 15) = 20.44. Each column represents the mean ± S.E.M. of *n* = 6 experiments performed in triplicate. * Significant versus all groups (*p* < 0.0001 versus CTL, *p* < 0.01 versus LC + GA); ** significant versus GA (*p* < 0.01). (**D**) F (2, 18) = 10.36. Each column represents the mean ± S.E.M. of *n* = 7 experiments performed in triplicate. * Significant versus all groups (*p* < 0.01 versus CTL, *p* < 0.05 versus LC + GA); ** significant versus GA and CTL (*p* < 0.05). Oligo = Oligomycin 3 µg/mL.

**Figure 3:**
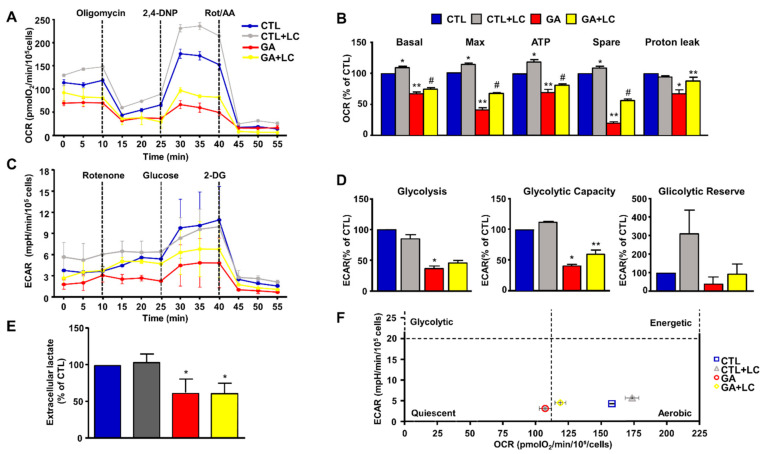
Energy metabolism characterization in rat cortical neurons challenged with GA and exposed to LC. Oxidative phosphorylation rate (**A**,**B**), glycolysis rate (**C**,**D**) and cell energy phenotype (**F**) were determined by Seahorse XF24 Extracellular Flux Analyzer. Glycolysis was also determined by using the Cayman Glycolysis Cell-Based Assay Kit (**E**). (**B**,**D**) Each column represents the mean ± S.E.M. of 3 replications. Statistical differences were assessed by one-way ANOVA followed by Dunnett’s post hoc test. (**E**) Each column represents the mean ± S.E.M. of *n* = 5 experiments performed in triplicate. Statistical differences were assessed by one-way ANOVA followed by Dunnett’s post hoc test. (**A**,**C**) Representative traces of both OCR and ECAR experiments. (**B**) Basal respiratory capacity: F (3, 8) = 144.7. * Significant versus all groups (*p* < 0.01 versus CTL, *p* < 0.0001 versus GA and LC + GA); ** significant versus all groups (*p* < 0.0001 vs. CTL and CTL + LC, *p* < 0.05 versus LC + GA); # significant versus all groups (*p* < 0.0001 vs. CTL and CTL + LC, *p* < 0.05 versus GA); maximal respiratory capacity: F (3, 8) = 357.5. * Significant versus all groups (*p* < 0.01 versus CTL, *p* < 0.0001 versus GA and LC + GA); ** significant versus all groups (*p* < 0.0001), # significant versus all groups (*p* < 0.0001); ATP: F (3, 8) = 63.24 * Significant versus all groups (*p* < 0.01 versus CTL, *p* < 0.0001 versus GA and LC + GA), ** significant versus all groups (*p* < 0.001 versus CTL, *p* < 0.0001 versus CTL + LC, *p* < 0.05 versus GA + LC), # significant versus all groups (*p* < 0.01 versus CTL, *p* < 0.0001 versus CTL + LC, *p* < 0.05 versus GA); spare respiratory capacity: F (3, 8) = 652.5. * Significant versus all groups (*p* < 0.01 versus CTL, *p* < 0.0001 versus GA and LC + GA), ** significant versus all groups (*p* < 0.0001), #significant versus all groups (*p* < 0.0001); proton leak: F (3, 8) = 10.50. * Significant versus all groups (*p* < 0.01 versus CTL and CTL + LC, *p* < 0.05 versus GA); ** significant versus GA (*p* < 0.05). (**D**) Glycolysis: F (3, 8) = 55.84. * Significant versus CTL and CTL + LC (*p* < 0.0001); glycolytic capacity: F (3, 8) = 101.2. * Significant versus all groups (*p* < 0.0001 versus CTL and CTL + LC, *p* < 0.05 versus GA + LC), ** significant versus all groups (*p* < 0.0001 versus CTL and CTL + LC, *p* < 0.05 versus GA). (**E**) F (3, 16) = 16.23. * Significant versus CTLs (*p* < 0.001). 2,4-DNP = 2,4-Dinitrophenol; Rot/AA = Rotenone/Antimycin A; 2-DG = 2-Deoxyglucose.

To elucidate the mechanisms underlying LC protection, we investigated the ability of this compound to affect overall cell metabolism on the background of GA challenged cells. We first analyzed ATP production. We explored the effect of LC on the intracellular ATP levels in SH-SY5Y cells and rat primary cortical neurons after 1 h of LC exposure under the control conditions. In this experimental setting, we found that LC exposure induced a significant increase in the intracellular ATP content (Figure 2A,B). Interestingly, LC-induced ATP generation completely relied on the oxidative phosphorylation process, since, in the presence of the ATP synthase inhibitor oligomycin (3 µg/mL) [15,40,42], ATP production was completely abolished (Figure 2A,B). When cells were pretreated with LC for 1 h and then exposed to GA for 24 h (without removing LC), the decrease in the ATP levels induced by the GA challenge was significantly blunted. This effect was observed in both SH-SY5Y cells and in rat primary cortical neurons (Figure 2C,D). Oligomycin did not significantly alter ATP production under the control conditions (data not shown). Considering the significant impact of GA on cell metabolism and ATP intracellular levels, we sought to further explore the effect of GA on energy management, by dissecting out its effect on glycolysis, measured as the extracellular acidification rate (ECAR), the L-lactate level secreted by cells, and mitochondrial respiration, assessed as the oxygen consumption rate (OCR). As reported in Figure 3, we found that in rat primary cortical neurons mitochondrial respiration (Figure 3A,B) and glycolysis (Figure 3C,D) were both significantly affected by the GA challenge. We observed that, in cells pretreated with LC and exposed to GA, LC positively affected mitochondrial respiration both under control conditions and in the presence of GA (Figure 3A,B). As shown in Figure 3B, on the one hand, LC rescued all the OCR parameters, including ATP (as previously observed, Figure 2D), on the other hand, LC did not show any significant effect on glycolysis and glycolysis reserves, but it significantly increased the glycolysis capacity (Figure 3D). As for glycolysis, the same result was obtained by measuring the L-lactate level (Figure 3E). Finally, the analysis of the cell energy phenotype showed that LC shifted the GA quiescent phenotype toward an aerobic phenotype, thereby increasing metabolic potential (Figure 3F).

### 3.3. LC Restored the Δψ_m_

**Figure 4:**
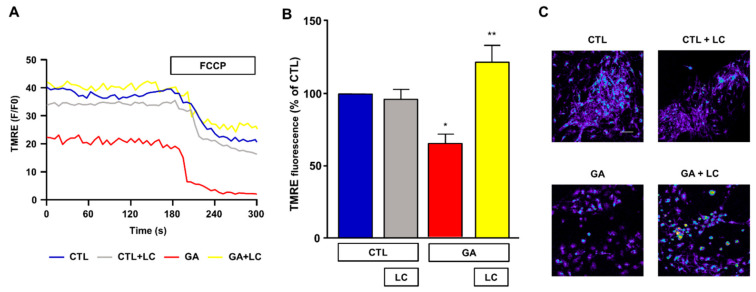
Analysis of mitochondrial membrane potential in rat primary cortical neurons challenged with GA and exposed to LC. Experiments were performed using a nonquenching concentration (10 nM) of the inner mitochondrial membrane potential indicator TMRE. In each experiment, FCCP (20 µM) was added at the end of the recording session as an internal control. (**A**) Representative records of mitochondrial membrane potential measurements in rat primary cortical neurons under control conditions (blue line), LC exposure (grey line), 24 h GA challenge (red line) and 24 h GA challenge in the presence of LC (yellow line). (**B**) Quantitative analysis of the mitochondrial membrane potential under the different experimental conditions. Each column represents the mean ± S.E.M. of 50–100 cells recorded in 4 different sessions (*n* = 4). In each experiment, TMRE fluorescence was expressed as percentage of the control. Statistical differences were assessed by one-way ANOVA followed by Dunnett’s post hoc test. (**C**) Representative images of mitochondrial membrane potential measurements. Scale bar 50 μm. (**B**) F (3, 12) = 9.53. * Significant versus all groups (*p* < 0.05 versus controls, *p* < 0.001 versus GA); ** significant versus GA (*p* < 0.001).

Mitochondrial respiration impairment and ATP depletion may be strictly correlated with the loss of the Δψ_m_, and, in this regard, a decreased Δψ_m_ was observed in AD animal models and in human cortical neurons ex vivo [9,10,11]. For this reason, we sought to investigate the effect of chronic GA treatment on the Δψ_m_ and evaluate any possible effect induced by LC. As shown in Figure 4, in rat primary cortical neurons, the GA challenge induced a significant decrease in the Δψ_m_. Notably, LC did not modify the Δψ_m_ under resting conditions, but it significantly inhibited the mitochondrial depolarization induced by GA exposure.

### 3.4. LC Scavenged Mitochondrial ROS in SH-SY5Y Cells and in Rat Primary Cortical Neurons Challenged with GA

**Figure 5:**
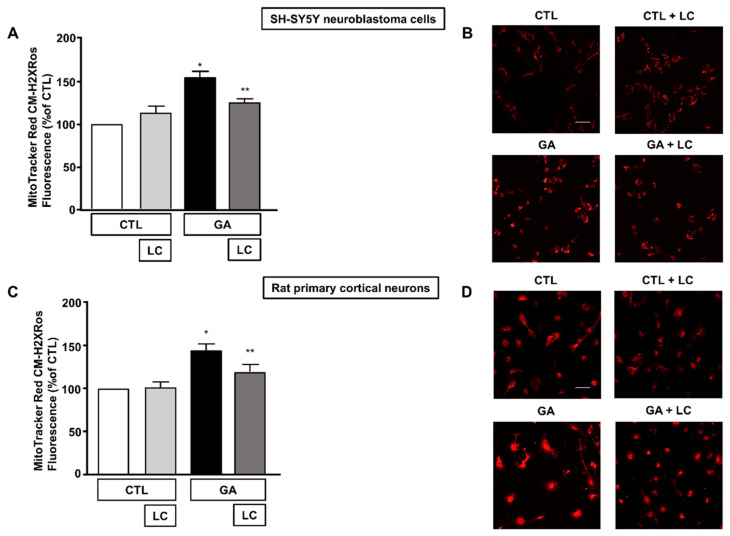
Effect of LC on mitochondrial ROS formation in SH-SY5Y neuroblastoma cells and in rat primary cortical neurons challenged with GA. (**A**,**C**) Quantitative analysis of MitoTracker Red CM-H2XRos fluorescence in SH-SY5Y neuroblastoma cells and in rat primary cortical neurons both exposed to 1 mM GA. Cells were pretreated with LC (3 mM) for 1 h and then exposed to GA (1 mM) for 24 h (without removing LC). (**B**,**D**) Representative images of mitochondrial ROS by MitoTracker Red CM-H2XRos staining. Images were representative of at least *n* = 6 independent experiments (50–100 cells for each experimental group were analyzed). Scale bar 50 μm. MitoTracker Red CM-H2XRos fluorescence intensity correlates with the levels of mitochondrial ROS. In each experiment, MitoTracker Red CM-H2XRos fluorescence was expressed as a percentage of the control. Statistical differences were assessed by one-way ANOVA followed by Dunnett’s post hoc test. (**A**) F (3, 20) = 19.01. * Significant versus all groups (*p* < 0.0001 versus controls and *p* < 0.01 versus LC + GA); ** significant versus GA and CTL (*p* < 0.01). (**C**) F (3, 21) = 10.01. * Significant versus all groups (*p* < 0.001 versus CTL, *p* < 0.01 versus CTL + LC, *p* < 0.05 versus LC + GA); ** significant versus GA (*p* < 0.05).

Alteration of the cell energy production and impairment of the Δψ_m_ can enhance the production of ROS by favoring improper electron transportation in the mitochondrial respiratory chain [10,50]. We have previously shown that GA can perturb the overall oxidative status of the cell [15], and several reports have described the ability of LC to act as a radical scavenger [30,33,34,35]. Therefore, we sought to explore whether LC could also manifest neuroprotective effects by preserving redox balance. In line with our specific interest, we tested the antioxidant properties of LC against the formation of ROS in mitochondria. When cells were pretreated with LC for 1 h and then exposed to GA for 24 h (without removing LC), mitochondrial ROS production significantly decreased (Figure 5). Interestingly, this effect was observed in both SH-SY5Y cells and in rat primary cortical neurons. LC did not significantly modify the mitochondrial ROS levels under the control conditions (Figure 5).

### 3.5. LC Significantly Attenuated the Increase in Intracellular pTau Levels Induced by GA Challenge

**Figure 6:**
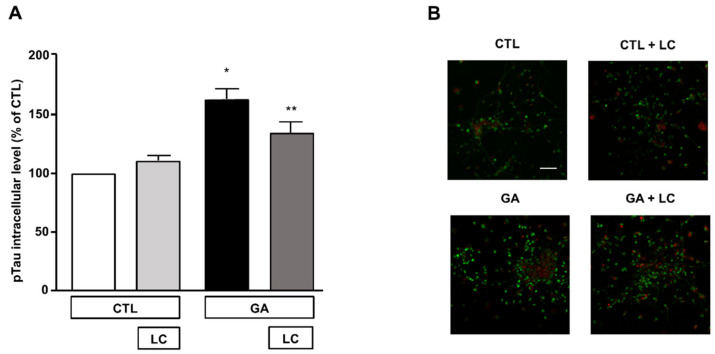
Analysis of pTau expression in rat primary cortical neurons challenged with GA and exposed to LC. (**A**) Quantitative analysis and (**B**) representative images of pTau expression in rat primary cortical neurons. The protein pTau was detected by immunofluorescence staining. Scale bar 50 μM. Each column represents the mean ± S.E.M. of *n* = 6 independent experiments (50–100 cells for each experimental group were analyzed). Differences among means were assessed by one-way ANOVA followed by Dunnett’s post hoc test. (**A**) F (3, 19) = 16.60. * Significant versus all groups (*p* < 0.0001 versus CTL, *p* < 0.001 versus CTL + LC, *p* < 0.05 versus LC + GA); ** significant versus GA (*p* < 0.05) and versus CTL (*p* < 0.01).

The formation of intracellular neurofibrillary tangles composed of pTau was one of the pathological hallmarks of AD [2,12]. A link between oxidative stress, mitochondrial dysfunction and the hyperphosphorylation of Tau has been described as a possible upstream event in AD and other Tau pathologies [12]. In view of this observation and in accordance with our experimental findings, we investigated the effect of LC on intracellular pTau levels, which are known to increase due to 24 h exposure to GA [15]. In the present experimental setting, we confirmed that the pTau levels increased after the GA challenge and found that LC was able to reduce this increase, as shown by the results of the immunofluorescence experiments reported in Figure 6. LC did not significantly modify intracellular pTau levels under the control conditions (Figure 6).

## 4. Discussion

In the present study, we demonstrated that, in SH-SY5Y neuroblastoma cells and in rat primary cortical neurons exposed to GA to compromise cell metabolism [15,36,51], LC significantly improved cell viability by enhancing their overall mitochondrial function. Specifically, LC sustained the ATP synthesis by stimulating mitochondrial respiration, counteracted the Δψ_m_ loss and reduced the free radical burden. Of note, LC caused a marked decrease in intracellular pTau levels, an effect that we hypothesize could contribute to promoting neuronal survival.

We recently reported that in different neuropathological settings, including AD, mitochondrial functions could be retained by supplying alternative substrates (e.g., glutamate) [15,40]. Glutamate could be used by neuronal cells to compensate for energy deficits and, at the same time, to scavenge for ROS, thus improving mitochondrial performance by acting at different sites [15,40]. In particular, in our in vitro model based on the alteration of cell metabolism [15], glutamate could prevent neuronal injury by increasing the intracellular ATP content, reducing both ROS formation and AD marker levels [15]. To further support our hypothesis, in the present study, we focused our attention on LC supplementation. LC is a naturally occurring compound found in the majority of mammalian tissues, including the brain [52]. Within cells, LC plays important functions in energy production since it facilitates the transport of activated long-chain fatty acids across the mitochondrial inner membrane so that they can be broken down through β-oxidation to acetate to produce energy via the Krebs cycle [16,52]. In addition, LC and its ester ALC may serve as sources of l-glutamate, further contributing to energy-producing reactions [35]. Consistent with this metabolic role, the therapeutic efficacy of LC was reported in infants affected by metabolic birth defects [53,54,55,56] and in patients with dilated cardiomyopathy [57], where LC, by stimulating the β-oxidation of fatty acids in cardiomyocytes, may improve myocardial function [58,59].

Several studies have provided evidence of the defects in the biosynthesis and metabolism of LC in AD patients [21,60,61,62], and low concentrations of LC and its derivatives were found in both plasma and tissues of AD patients [20,21,60]. In particular, a progressive decrease in LC derivatives was observed in the serum of subjects affected by increasingly severe cognitive decline up to AD, in comparison with those in healthy subjects [20]. Subsequently, several studies have reported that the administration of LC derivatives can improve cognitive performance and slow mental decline, especially in patients displaying mild cognitive impairment (a transition state between normal brain aging and AD [63]) and in early-stage or mildly affected AD patients [22,24,26].

A clear picture of the mechanisms underlying the beneficial effects of LC cannot be drawn yet, but available data suggest that multiple factors may be involved [64,65]. In our experimental setting, we found that in both SH-SY5Y neuroblastoma cells and in rat primary cortical neurons, pre-exposure to LC significantly improved cell viability, which was compromised by the GA challenge at different sites. We demonstrated that GA produced a profound alteration of cell metabolism, by affecting both glycolysis and mitochondrial respiration, leading to a dramatic decrease in intracellular ATP content. The alteration of neuronal metabolism has been recognized as a possible upstream event of the degenerative processes leading to AD [4,5] and a link with the increase in AD marker levels has been hypothesized for the GA challenge [6,36]. There is evidence that GA-induced alteration in AD marker levels may also rely on its ability to favor ROS formation through the production of AGEs [7,66,67,68]. Similarly, we have previously found that GA exposure can increase the levels of Aβ_1–42_, pTau and mitochondrial ROS in retinoic acid-differentiated SH-SY5Y and rat primary cortical neurons [15], supporting the hypothesis that hypometabolism-correlated alterations may converge on a common path leading to AD.

Here, we found that in cells pretreated with LC for 1 h and then exposed to GA for 24 h, LC significantly rescued the overall intracellular ATP levels. Of note, LC pretreatment significantly stimulated the production of ATP, suggesting that the improvement of cell energy status may drive the overall cell viability rescue in such a metabolic stress situation [63,69]. It is worth noting that LC promoted the generation of ATP via oxidative phosphorylation, since, in the presence of the F1F0 ATP synthase inhibitor, oligomycin, LC did not stimulate any increase in intracellular ATP levels. Accordingly, we found that mitochondrial respiration, dramatically compromised by the GA challenge, was significantly stimulated in the presence of LC, which showed the ability to elicit an increase in the mitochondrial oxidative phosphorylation rate, per se. Interestingly, while GA significantly inhibited glycolysis, LC was ineffective in restoring this metabolic pathway. LC may support brain energy homeostasis and ATP synthesis by supplying acyl groups to mitochondria [60] or, alternatively, it is tempting to speculate the involvement of mitochondrial biogenesis [70]. Regardless of the mechanism, LC can be seen as an alternative source of energy under the condition of a glycolytic flow reduction, which is often observed in early-stage AD [71,72]. In summary, neurons pretreated with LC seem to be better equipped to face a condition of increased energy need, as confirmed by the increase in the spare respiratory capacity, which reflects the ability to produce more ATP and to maintain adequate levels of energetic molecules to overcome stressful situations. Our bioenergetic analysis performed in cortical neurons also revealed significant changes in proton leak, which could be explained by considering the alteration of the Δψ_m_. Under control conditions, the OCR, as a function of Δψ_m_, increased exponentially [73], and this condition tended to generate a mitochondrial potential which favored the entry of H^+^ into the matrix [73]. In this view, proton leak may contribute to the maintenance of the cellular metabolic rate and the steady proton gradient, thereby regulating Δψ_m_ and mitochondrial integrity [74]. Thus, Δψ_m_, OCR (and consequently ATP) and proton leak are closely related: sustained changes in one of these factors may be detrimental for mitochondrial stability [74]. In our setting, as OCR (and ATP) decreased, we observed a reduction of the proton leak, reflecting a depolarization of the mitochondrial membrane, which probably impaired the H^+^ influx into the matrix, thereby destabilizing mitochondrial functions. We can speculate that by sustaining aerobic energy metabolism, LC contributed to restoring this delicate balance. In addition, based on the existing literature, we can also hypothesize that the stabilization of the mitochondrial membrane potential may also rely on the increase in the synthesis of phospholipids, which are required to ensure proper membrane formation and integrity [75,76].

Several reports also describe that LC and its acyl derivative ALC exert neuroprotective effects by means of their antioxidant properties [28,30,33,34,35]. Therefore, we explored this possible effect in our experimental setting. As mentioned above, GA exposure can significantly increase the formation of ROS at both cytoplasmic and mitochondrial levels [15]. Considering that our results suggested the ability of LC to ameliorate mitochondrial function, we specifically verified its effects on mitochondrial ROS production. As expected, in both SH-SY5Y neuroblastoma cells and in rat primary cortical neurons, GA exposure significantly increased the formation of ROS in mitochondria. Supplementation with LC 1 h before the addition of GA and maintaining LC during the entire period of GA exposure, significantly reduced mitochondrial ROS formation, confirming that the neuroprotective action of LC may also have relied on its antioxidant properties. In the present study, we did not investigate further the actual mechanisms leading to the antioxidant effects of LC; however, several mechanisms may play a role. For instance, it has been proposed that LC can prevent free radical production by inhibiting the activity of enzymes involved in the generation of free radicals and by inducing antioxidant mechanisms, including the activity of key enzymes, such as superoxide dismutase (SOD) [35,76]. A relationship between ROS and the proton leak has also been described and extensively debated. Under resting physiological conditions, a stable H^+^ leak has been proposed to be beneficial, by decreasing ROS generation [77,78]. However, other reports indicate that mitochondrially generated ROS could increase proton leak [79], but in return, increased proton leak may suppress ROS production, suggesting the existence of a positive feedback loop that could protect the biological systems from the deleterious effects of augmented oxidative stress [78,80]. Since LC rescued the GA-induced reduction of proton leak, we speculated that it could be the driving force behind this positive feedback.

The specific mechanisms that provide a link between hypometabolism and AD are still matter of intense research. Recent findings suggest that oxidative stress may be a crucial factor in determining whether Tau phosphorylation contributes to a neurodegenerative process rather than to sustaining the intracellular structures that grant neuronal functions [12]. In line with this finding, it is possible to hypothesize that interventions which can reduce oxidative stress can also affect the pTau level, thus contributing to neuronal health. As we have previously observed, the GA challenge significantly increased the intracellular level of pTau in rat primary cortical neurons. [15]. In the present experimental setting, a significant reduction in intracellular pTau levels was observed in neurons pretreated with LC. Although we did not perform specific experiments, we can speculate that the decrease in Tau phosphorylation induced by LC could be a downstream effect related either to its antioxidant effect or to its metabolic role. For instance, in an in vitro model based on M17 neuroblastoma cells, in which the synthesis of glutathione was inhibited, increased levels of phosphorylated Tau were observed [81]. In null mice lacking SOD, characterized by mitochondrial dysfunction and oxidative stress, an abnormal phosphorylation of Tau was also found [82]. In this framework, alterations of glucose metabolism (as those observed in diabetes) may also have a role. Increased levels of phosphorylated Tau were observed in different settings, including db/db mice, streptozotocin-treated wild type mice, and, importantly, in human diabetic brains [83,84,85,86,87]. Notably, the antidiabetic drug metformin was shown to reduce the levels of phosphorylated Tau both in vitro and in vivo [87], supporting the hypothesis that glucose-energy metabolism and Tau modulation may be closely related. Considering the interplay between oxidative stress and dysfunctional glucose metabolism in AD [4], we cannot rule out that GA may establish a vicious cycle that feeds Tau abnormal phosphorylation. Further investigations are needed to elucidate this process as well as the exact role of LC in this context.

Overall, we propose that alterations of the cellular metabolic pathways, as obtained in our setting via GA exposure, may trigger a bioenergetics crisis that alters mitochondrial homeostasis and shifts redox balance towards the overproduction of ROS and the loss of the Δψ_m_, whose stability, together with ATP generation, is crucial for the maintenance of mitochondrial homeostasis. These effects are paralleled by an increase in AD marker levels. In this scenario, LC acts at different sites: (1) by compensating for the reduction in energy metabolism induced by GA through the stimulation of ATP synthesis; consequently (2) by counteracting the mitochondrial membrane depolarization induced by GA; and (3) by improving neuronal antioxidant defenses. In this context, the improvement of mitochondrial function and the restoration of metabolic homeostasis appears to contribute to shifting the balance towards a reduction in Tau phosphorylation, further supporting neuronal health. Despite these advantages, it should be noted that LC does not guarantee a full restoration of neuronal vitality. On the one hand, LC is able to reduce the intracellular levels of pTau; on the other hand, it does not seem to have a significant effect on the level of the amyloid precursor protein (data not shown) and, consequently, the level of Aβ. This observation strengthens the concept that multiple molecular mechanisms are involved in AD and that a multifunctional approach may be required to effectively counteract neurodegeneration. Considering the central role played by the cellular bioenergetics in the present experimental setting, we cannot rule out that the partial efficacy of LC may be related to the absence of the astrocytic component. Since our cellular model is essentially based on neurons, at present we have no clear information on the possible contribution of astrocytes, which are known to affect the overall functions of surroundings neurons, both in heath and disease [88,89]. In this view, further research efforts are required to shed more light on the role of astrocytes in our experimental conditions.

In conclusion, the present study suggests that LC could be protective against GA-induced metabolic impairment. Our data support the importance of promoting therapeutic strategies that could act on early pathological events rather than on their consequences, thereby ameliorating neuronal survival in the neurodegenerative context that characterizes AD.

## Data Availability

The data presented in this study are available on request from the corresponding author.
